# Design, Synthesis, and Antimicrobial Activity of Certain New Indole-1,2,4 Triazole Conjugates

**DOI:** 10.3390/molecules26082292

**Published:** 2021-04-15

**Authors:** Reem I. Al-Wabli, Mona A. Alsulami, Sarah I. Bukhari, Nadine M. S. Moubayed, Maha S. Al-Mutairi, Mohamed I. Attia

**Affiliations:** 1 Department of Pharmaceutical Chemistry, College of Pharmacy, King Saud University, P.O. Box 2457, Riyadh 11451, Saudi Arabia; aa_88_22@hotmail.com (M.A.A.); malmutbiri@ksu.edu.sa (M.S.A.-M.); 2 Department of Pharmaceutics, College of Pharmacy, King Saud University, P.O. Box 2457, Riyadh 11451, Saudi Arabia; Sbukhari@ksu.edu.sa; 3 Botany and Microbiology Department, College of Science, King Saud University, Riyadh 11451, Saudi Arabia; nmoubayed@ksu.edu.sa; 4 Medicinal and Pharmaceutical Chemistry Department, Pharmaceutical and Drug Industries Research Division, National Research Centre (ID: 60014618), El Bohooth Street, Dokki, Giza 12622, Egypt

**Keywords:** indole, 1,2,4-triazole-3-thiol, antibacterial, antifungal

## Abstract

The increasing prevalence of microbial infections and the emergence of resistance to the currently available antimicrobial drugs urged the development of potent new chemical entities with eminent pharmacokinetic and/or pharmacodynamic profiles. Thus, a series of new indole-triazole conjugates **6a-u** was designed and synthesized to be assessed as new antimicrobial candidates using the diameter of the inhibition zone and minimum inhibitory concentration assays against certain microbial strains. Their in vitro antibacterial evaluation revealed good to moderate activity against most of the tested Gram-negative strains with diameter of the inhibition zone (DIZ) values in the range of 11–15 mm and minimum inhibition concentration (MIC) values around 250 µg/mL. Meanwhile, their in vitro antifungal evaluation demonstrated a potent activity against *Candida tropicalis* with MIC value as low as 2 µg/mL for most of the tested compounds. Moreover, compound **6f** is the most potent congener with an MIC value of 2 µg/mL against *Candida albicans*.

## 1. Introduction

Bacterial infections are the major cause of some high mortality rate diseases such as typhus, tuberculosis, plague, diphtheria, cholera, dysentery, and pneumonia. On the other hand, fungi infect many people worldwide every year, yet their contribution to the global burden of diseases is largely unrecognized, and most of them cause relatively minor infections but kill at least as many people as tuberculosis or malaria [1]. This is due to an increasing population with reduced immune system or immunocompromised individuals having AIDS, cancer or those undergoing organ transplantation. Furthermore, microbial infections are becoming more resistant to antibiotics due to years of their overuse and/or misuse, which might lead to a potential global health disaster [2]. In addition, the current hospital-acquired infections are resistant to most of the clinically available antibiotics such as methicillin and vancomycin. Therefore, the use of these drugs was reserved to treat only the most intractable infections to retard the development of their resistance [3,4,5]. This makes the design and development of new antimicrobial candidates with novel chemical structures and/or with different modes of action rather than analogues of the existing ones are necessary for clinical needs.

Indole ring is one of the most potent pharmacodynamic nucleus and constitutes the backbone of the neurotransmitter serotonin and the natural hormone melatonin as well as the essential amino acid tryptophan. The recognition of tryptophan importance in animal and human nutrition served to bring about a renaissance in indole chemistry, and therefore, indole derivatives have been synthesized and/or isolated from nature in abundance. Mitomycin is an example of bioactive indole alkaloids and its analogues are gaining great attention because of their extensive use in cancer chemotherapy and for its antibacterial activity [6,7]. Consequently, compounds bearing an indole nucleus have a considerable importance in the development of diverse bioactive compounds by varying the substituents or substitution pattern of the indole ring. Such compounds have been reported for various biological activities including antimicrobial [8], antiviral [9], anticonvulsant [10], analgesic, anti-inflammatory anti-tubercular [11] and anticancer [12]. Thus, this wide spectrum of activities of indole-bearing compounds makes them suitable candidates for the development of novel antibacterial and antifungal treatments. On the other hand, there has been a considerable interest in the development of novel heterocyclic compounds with variable biological activities. Among them, triazoles are a special class of heterocyclic compounds with a broad spectrum of biological activities such as antibacterial [13,14], antifungal [15,16], anti-inflammatory [17], antitubercular [18], anticancer [19], antimalarial [20], antiviral [21], and analgesic [22]. Furthermore, fluconazole and itraconazole [23] are therapeutically significant antifungal azoles containing 1,2,4-triazole moiety.

Hybridization is an important modality in drug design and development based on a combination of biologically active moieties to produce a new hybrid compound with an improved biological activity when compared to the parent compounds. Additionally, this strategy can result in compounds presenting a modified selectivity profile, different and/or dual modes of action, and reduced undesired side effects [24]. A literature review revealed that 1,2,4 triazole-indole hybrid compounds were synthesized and demonstrated excellent antibacterial and antifungal activity [25]. In addition, it was found that a free N–H in the indole ring was essential and increased the antibacterial activity to an acceptable extent against *K. pneumoniae*, *E. coli*, and *P. aeruginosa* [26]. Moreover, the 1,2,4-triazole nucleus has received attention due to its low toxicity, wide safety, and good pharmacokinetic characteristics [27], and it was noticed that upon the introduction of the electron-rich sulfur atom into the triazole ring, the bioactivities of the target compounds greatly improve [28]. This may be attributed to the effect of the sulfur atom in increasing the lipophilicity and modulating of the electron density in the triazole ring, which increases the transmembrane diffusion and their interaction with macromolecular targets [29].

On the basis of the aforementioned premises, it was of our interest to design and synthesize certain indole-based 1,2,4-triazole-3-thiol molecular hybrids **6a-u** with different N_4_ substituent on the triazole nucleus with increasing order of lipophilicity (cyclohexyl < phenyl < 4-Cl-phenyl) and to test their antibacterial and antifungal activities.

## 2. Results and Discussions

### 2.1. Chemistry

**Scheme 1:**
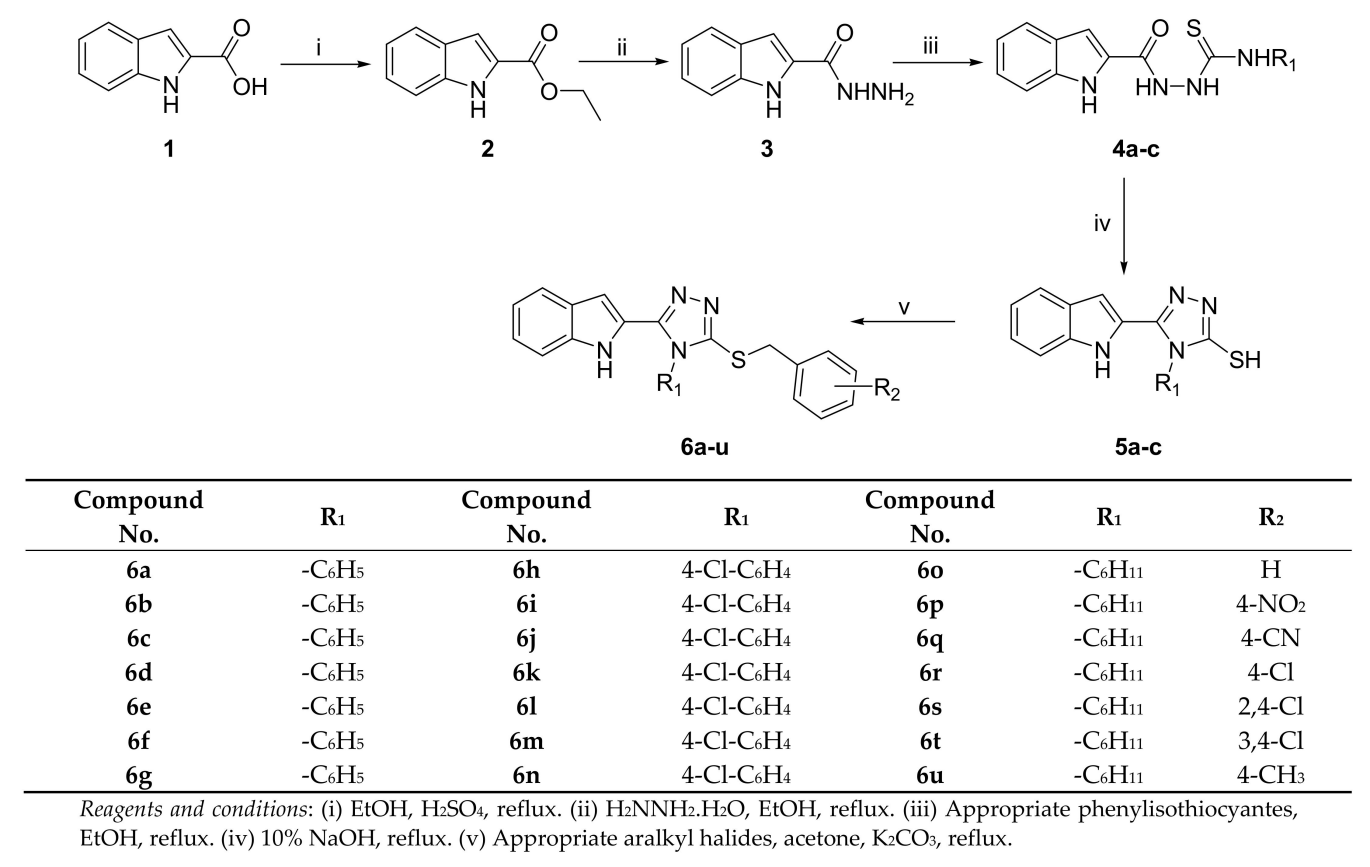
Synthesis of the target compounds **6a-u**.

The target compounds **6a-u** have been successfully synthesized according to Scheme 1. The synthesis was commenced by esterification of the commercially available indole-2-carboxylic acid (**1**) in ethanol in the presence of a catalytic amount of sulfuric acid. The ethyl ester derivative **2** was converted to the corresponding acid hydrazide **3** through reaction with hydrazine hydrate in ethanol. Subsequently, the appropriate isothiocyanate, namely cyclohexyl isothiocyanate, phenyl isothiocyanate, and 4-chlorophenyl isothiocyanate was allowed to react with compound **3** to yield the respective thiosemicarbazide derivatives **4a-c**. Cyclodehydration reaction of the thiosemicarbazide derivatives **4a-c** was carried out in aqueous 10% NaOH to give compounds **5a-c**. Thereafter, the target compounds **6a-u** were achieved through a nucleophilic substitution reaction in which the appropriate benzyl halide was subjected to nucleophilic attack by the thiol group of compounds **5a-c**. It is noteworthy to mention that the use of polar protic solvents such as ethanol, in a strong alkaline medium such as sodium hydroxide, favors the formation of both *S*-alkyl derivatives as major products and *N*-alkyl derivatives as minor products. On the other hand, carrying out the reaction in a polar aprotic solvent such as acetone, in the presence of potassium carbonate favors the formation of *S*-alkyl derivatives as a sole product [30]. Thus, the triazole derivatives **5a-c** were elaborated to their respective *N*-alkyl derivatives **6a-u** as sole products through the reaction with the appropriate benzyl halide in dry acetone [31]. The ^1^H-NMR spectra of compounds **6a-u** showed the cyclohexyl protons for compounds **6o-u** in the range of δ = 1.05-4.40 ppm. Meanwhile, compounds **6g**, **6n**, and **6u** showed singlets at δ = 2.28 ppm representing three protons of CH_3_ group. Singlets integrated for two protons were noticed in the range of δ = 4.36-4.70 ppm, which were assigned to be for the two protons of the benzylic CH_2_. The aromatic protons were observed in the region of δ = 6.94-8.19 ppm. Additionally, the SH signals disappeared, and the ^13^C-NMR spectra of the target compounds **6a-u** exhibited signals in the range of δ = 34.7-37.3 ppm indicating carbons of the benzylic CH_2_. The cyclohexyl carbons for compounds **6o-u** were observed in the range of δ = 24.9-56.8 ppm and the aromatic carbons appeared in the range of δ = 101.8-151.7 ppm.

### 2.2. Antimicrobial Evaluation

The in vitro antibacterial potential of the tested compounds **6a-u** was estimated against five standard bacterial strains, namely the Gram-negative *Escherichia coli*, *Pseudomonas aeruginosa,* and *Salmonella*, and the Gram-positive *Staphylococcus aureus* and *Bacillus subtilis*. Meanwhile, the antifungal potential was estimated against three standard fungal strains, namely *Candida albicans*, *Candida tropicalis,* and *Candida glabrata*. The results are expressed as diameter of the inhibition zone (DIZ) and the minimum inhibition concentration (MIC). Ampicillin (antibacterial) and fluconazole (antifungal) were used as reference drugs, and the antimicrobial results are presented in Table 1 and Table 2.

Table 1 illustrates the antibacterial activity of the target compounds **6a-u** in the DIZ and MIC assays against the tested bacterial strains. They showed varying degrees of activities against the tested microorganisms. Most of the investigated compounds **6a-u** manifested good to moderate activity in the DIZ assay toward the tested Gram-negative bacterial strains with DIZ values in the range of 11-15 mm. Whereas, their MIC value was around 250 µg/mL which is lower than the MIC values of ampicillin against *Escherichia coli* (500 µg/mL), *Pseudomonas aeruginosa* (1000 µg/mL), and *Salmonella* (500 µg/mL). In addition, Gram-positive bacteria were less sensitive toward the tested compounds **6a-u** than Gram-negative bacteria. Usually, increasing the lipophilic characteristic of the synthesized compound increases the antibacterial activity on Gram-positive bacteria. This can be explained on the bases of the low lipid content of the cell wall of Gram-positive bacteria, which would permit the activity of more lipophilic compounds [32]. Only compounds **6h-n**, bearing the 4-chlorophenyl moiety, exhibited good activity against the tested standard *Staphylococcus aureus* as it showed MIC values of 250-1000 µg/mL. Furthermore, compounds **6b-g** bearing phenyl moiety on the triazole ring showed MIC values of 250-500 µg/mL against *Bacillus subtilis* being similar to or better than the MIC value of the reference drug ampicillin (500 µg/mL). In contrast, compounds with *N*-4-chlorophenyl or *N*-cyclohexyl substituents did not show activity against *Bacillus subtilis* in the DIZ assay (Table 1). It was noticed that in general, the unsubstituted benzyl derivatives **6a**, **6h**, and **6o** exhibited lower antibacterial activity against both Gram-negative and Gram-positive bacteria, which indicates the importance of the para substitution in the benzyl moiety.

The in vitro antifungal activity of the target compounds **6a-u** is presented in Table 2. They displayed potent in vitro antifungal activity against *Candida tropicalis* with an MIC value as low as 2 µg/mL. Moreover, compounds **6a-g** bearing *N*-phenyl substituent showed a DIZ value around 27 mm against the tested *Candida albicans* strain. Most of compounds **6a-g** exhibited MIC value of 250 µg/mL being equal to that of the reference drug fluconazole except for compound **6f** bearing 3, 4-dichlorbenzyl moiety, which revealed a potent MIC value of 2 µg/mL. Meanwhile, compounds **6o-u** with *N*-cyclohexyl substituent displayed moderate to good activity against the tested *Candida albicans* strain, in which compounds **6p**, **6q**, and **6r** are the most active congeners with an MIC value of 32 µg/mL. In addition, compounds **6a-g** exhibited MIC values of 125-1000 µg/mL against *Candida glabrata* in which compound **6d** was the most active candidate. Furthermore, compounds **6s** (bearing 2,4-dichlorobenzyl and *N*-cyclohexyl substituents), **6t** (bearing 3,4-dichlorobenzyl and *N*-cyclohexyl substituents), and **6u** (bearing 4-methylbenzyl and *N*-cyclohexyl substituents) manifested good activity toward *Candida glabrata* with MIC values of 62.5, 32, and 16 µg/mL, respectively. Unexpectedly, the presence of 4-chlorophenyl moiety on the triazole ring of compounds **6h-n** seems to be unfavorable for their activities against both *Candida albicans* and *Candida glabrata*. In addition, the influence of the *para* benzyl substituent in increasing the antifungal activity can be detected from the lower antifungal activity of the unsubstituted benzyl derivatives **6a** and **6b**.

The in vitro antifungal activity of the target compounds **6a-u** against most of the tested fungal strains was generally higher than their antibacterial activity. This could be attributed to the presence of the triazole ring in their scaffold, which is consistent with the presence of the triazole ring in a number of clinically used antifungal agents [33].

## 3. Experimental

### 3.1. General

The melting points were measured using a Gallenkamp melting point device and are uncorrected. The NMR samples of the synthesized compounds **6a-u** were dissolved in DMSO-d6 and the NMR spectra were recorded using a Bruker NMR spectrometer (Bruker, Reinstetten, Germany) at 500 MHz for ^1^H and 125.76 MHz for ^13^C at the Research Center, College of Pharmacy, King Saud University, Saudi Arabia. TMS was used as an internal standard, and chemical shift values were recorded in ppm on the δ scale. The ^1^H NMR spectral data are represented as follows: chemical shifts, multiplicity (s, singlet; d, doublet; t, triplet; m, multiplet) and number of protons. The ^13^C NMR spectral data were represented as chemical shifts and type of carbon. Mass spectra were measured on an Agilent Quadrupole 6120 LC/MS with ESI (electrospray ionization) source (Agilent Technologies, Palo Alto, CA, USA). Elemental analysis was carried out at the Microanalysis Laboratory, Cairo University, Cairo, Egypt, using an Elemental C, H, N analyzer Vario EL III, Germany, and the results agreed favorably with the proposed structures within ± 0.4% of the theoretical values. Silica gel thin layer chromatography (TLC) plates from Merck (silica gel precoated aluminium plates with fluorescent indicator at 254 nm) were used for thin layer chromatography. Visualization was performed by illumination with a UV light source (254 nm). The microanalysis data, ^1^HNMR and ^13^CNMR of compound **6d** are also available in the Supplementary Materials.

### 3.2. Chemistry

#### 3.2.1. Synthesis of Ethyl 1*H*-Indole-2-Carboxylate (**2**)

1*H*-indole-2-carboxylic acid was subjected to esterification in absolute ethanol and a catalytic amount of sulfuric acid according to the reported method. The crude ester **2** was used in the next step without further purification [34,35].

#### 3.2.2. Synthesis of 1*H*-Indole-2-Carbohydrazide (**3**)

Compound **2** (0.875 g, 5 mmol) was suspended in absolute ethanol (10 mL), and hydrazine hydrate (2.5 mL, 50 mmol) was added. The reaction mixture was subjected to reflux for six hours under stirring. The reaction mixture was cooled to ambient temperature. The precipitated solid was filtered and dried to afford the carbohydrazide **3 [34,36]**.

#### 3.2.3. General Procedure for the Synthesis of the Indole-1*H*-2-yl-4-Substituted-Thiosemicarbazides (**4a**-**c**)

A mixture of 1*H*-indole-2-carbohydrazide (**3**, 6 mmol) and the appropriate isothiocyanate (6 mmol) in absolute ethanol (10 mL) was heated under reflux for five hours, cooled, the precipitated solid was filtered off and dried to give the thiosemicarbazides **4a-c [34]**, which were used without further purification [31].

#### 3.2.4. General Procedure for the Synthesis of 5-(1*H*-Indol-2-yl)-4-Substituted-1,2,4-Triazole-3-Thiols (**5a**-**c**)

Aqueous 10% NaOH (3.3 mL) was added to the appropriate thiosemicarbazide derivative **4a-c** (10 mmol). The reaction mixture was heated to reflux for three hours, cooled, and neutralized with 5N HCl. The precipitated products **5a-c** [34] were collected by filtration and re-crystallized from ethanol [31].

#### 3.2.5. General Procedure for the Synthesis of the Target Compounds **6a**-**u**

The appropriate benzyl halide (1.1 mmol) was added dropwise to a stirred suspension of the appropriate triazole derivative **5a-c** (1 mmol) and anhydrous potassium carbonate (1.1 mmol) in dry acetone (15 mL). The reaction mixture was heated to reflux under stirring for two hours. The corresponding alkylated indoles **6a-u** were collected by filtration and recrystallized from ethanol [37].

*2-(5-(Benzylthio)-4-phenyl-4H-1,2,4-triazol-3-yl)-1H-indole* (**6a**) [34]. White powder m.p. 250 °C (yield 99%); IR (KBr): ν (cm^−1^) 3163 (NH), 3061 (C-H, aromatic), 2980 (C-H, aliphatic), 1577, 1570 (C=N), 1494, 1450, 701; ^1^H NMR (DMSO-*d*_6_) *ppm*: 4.42 (s, 2H, CH_2_), 5.58 (d, *J* = 2.1 Hz, 1H, H-3), 6.94-6.96 (m, 1H, H_ar_.), 7.14-7.15 (m, 1H, H_ar_.), 7.27-7.29 (m, 1H, H_ar_.), 7.30-7.31 (m, 2H, H_ar_.), 7.34-7.36 (m, 3H, H_ar_.), 7.39-7.40 (m, 2H, H_ar_.), 7.43-7.44 (m, 1H, H_ar_.), 7.62-7.64 (m, 2H, H_ar_.), 7.67-7.69 (m, 1H, H_ar_.), 12.01 (s, 1H, NH); ^13^C NMR (DMSO-*d*_6_) *ppm*: 37.11 (-S-CH_2_), 101.8, 112.3, 120.2, 121.2, 123.6, 124.3, 127.6, 128.0, 128.4, 128.9, 129.5, 130.6, 131.1, 134.1, 136.9, 137.4, 149.6, 151.7 (C_ar_., CH_ar_., CN); MS *m/z* (ESI): 383.2 [M + H]^+^.

*2-(5-(4-Nitrobenzylthio)-4-phenyl-4H-1,2,4-triazol-3-yl)-1H-indole* (**6b**). Yellow powder m.p. 271 °C (yield 90%); IR (KBr): ν (cm^−1^) 3147 (NH), 3061 (C-H, aromatic), 2987 (C-H, aliphatic), 1597, 1590 (C=N), 1516, 1338 (NO_2_), 1490, 1309, 800; ^1^H NMR (DMSO-*d*_6_) *ppm*: 4.55 (s, 2H, CH_2_), 5.59 (s, 1H, H-3), 6.94-6.96 (m, 1H, H_ar_.), 7.13-7.15 (m, 1H, H_ar_.), 7.35 (d, *J* = 7.7 Hz, 1H, H_ar_.), 7.43-7.44 (m, 1H, H_ar_.), 7.48 (d, *J* = 7.7 Hz, 2H, H_ar_.), 7.64-7.70 (m, 5H, H_ar_.), 8.18 (d, *J* = 8.4 Hz, 2H, H_ar_.), 11.99 (s, 1H, NH); ^13^C NMR (DMSO-*d*_6_) *ppm*: 35.8 (-S-CH_2_), 101.9, 112.3, 120.2, 121.2, 123.6, 124.0, 124.2, 127.6, 128.4 130.7, 130.8, 131.2, 134.0, 136.9, 146.0, 147.1, 149.8, 151.3 (C_ar_., CH_ar_.); MS *m/z* (ESI): 428.1 [M+H]^+^.

*4-((5-(1H-Indol-2-yl)-4-phenyl-4H-1,2,4-triazol-3-ylthio)methyl)benzonitrile* (**6c**). White powder m.p. 252 °C (yield 90%); IR (KBr): ν (cm^−1^) 3273 (NH), 3100 (C-H, aromatic), 2980 (C-H, aliphatic), 2227 (CN), 1595, 1590 (C=N), 1494, 1450, 700; ^1^H NMR (DMSO-*d*_6_) *ppm*: 4.49 (s, 2H, CH_2_), 5.59 (d, *J* = 1.4 Hz, 1H, H-3), 6.94-6.96 (m, 1H, H_ar_.), 7.14 (t, *J* = 7.7 Hz, 1H, H_ar_.), 7.35 (d, *J* = 8.4 Hz, 1H, H_ar_.), 7.44 (d, *J* = 8.4 Hz, 1H, H_ar_.), 7.47 (d, *J* = 8.4 Hz, 2H, H_ar_.), 7.59 (d, *J* = 8.4 Hz, 2H, H_ar_.), 7.64-7.66 (m, 2H, H_ar_.), 7.68-7.70 (m, 1H, H_ar_.), 7.79 (d, *J* = 8.4 Hz, 2H, H_ar_.), 11.99 (s, 1H, NH); ^13^C NMR (DMSO-*d*_6_) *ppm*: 36.2 (-S-CH_2_), 101.9, 110.6, 112.3, 119.2, 120.2, 121.2, 123.6, 124.2, 127.6, 128.4, 130.5, 130.7, 131.2, 132.8, 134.0, 136.9, 143.8, 149.8, 151.4 (C_ar_., CH_ar_., CN); MS *m/z* (ESI): 405.9 [M-H]^−^.

*2-(5-(4-Chlorobenzylthio)-4-phenyl-4H-1,2,4-triazol-3-yl)-1H-indole* (**6d**). White powder m.p. 257 °C (yield 90%); IR (KBr): ν (cm^−1^) 3242 (NH), 3100 (C-H, aromatic), 2808 (C-H, aliphatic), 1600, 1597 (C=N), 1400, 1195, 700; ^1^H NMR (DMSO-*d*_6_) *ppm*: 4.41 (s, 2H, CH_2_), 5.58 (d, *J* = 1.4, 1H, H-3), 6.94-6.96 (m, 1H, H_ar_.), 7.13-7.15 (m, 1H, H_ar_.), 7.35 (d, *J* = 8.4, 1H, 1H, H_ar_.), 7.37-7.40 (m, 4H, H_ar_.), 7.43-7.45 (m, 3H, H_ar_.), 7.65 (t, *J* = 7.7 Hz, 2H, H_ar_.), 7.68-7.70 (m, 1H, H_ar_.), 11.98 (s, 1H, NH); ^13^C NMR (DMSO-*d*_6_) *ppm*: 36.0 (-S-CH_2_), 101.9, 112.3, 120.2, 121.2, 123.6, 124.3, 127.6, 128.4, 128.9, 130.7, 131.2, 131.4, 132.6, 134.1, 136.8, 136.9, 149.7, 151.6 (C_ar_., CH_ar_.); MS *m/z* (ESI): 415.7 [M-H]^−^, 417 [(M + 2)-H]^−^.

*2-(5-(2,4-Dichlorobenzylthio)-4-phenyl-4H-1,2,4-triazol-3-yl)-1H-indole* (**6e**). White powder m.p. 255 °C (yield 99%); IR (KBr): ν (cm^−1^) 3250 (NH), 3111 (C-H, aromatic), 2935 (C-H, aliphatic), 1600, 1597 (C=N), 1496, 1398, 800; ^1^H NMR (DMSO-*d*_6_) *ppm*: 4.45 (s, 2H, CH_2_), 5.59 (s, 1H, H-3), 6.94-6.96 (m, 1H, H_ar_.), 7.13-7.15 (m, 1H, H_ar_.), 7.35 (d, *J* = 7.7 Hz, 1H, H_ar_.), 7.41 (dd, *J* = 7.7, 2.1 Hz, 1H, H_ar_.), 7.43 (d, *J* = 7.7 Hz, 1H, H_ar_.), 7.46 (d, *J* = 7 Hz, 2H, H_ar_.), 7.54 (d, *J* = 8.4 Hz, 1H, H_ar_.), 7.64-6.66 (m, 3H, H_ar_.), 7.69 (t, *J* = 7.7 Hz, 1H, H_ar_.), 12.00 (s, 1H, NH); ^13^C NMR (DMSO-*d*_6_) *ppm*: 34.7 (-S-CH_2_), 101.9, 112.3, 120.2, 121.2, 123.7, 124.2, 127.6, 127.9, 128.4, 129.5, 130.7, 131.2, 133.2, 133.7, 134.0, 134.2, 134.7, 136.9, 149.9, 150.9 (C_ar_., CH_ar_.); MS *m/z* (ESI): 448.8 [M-H]^−^, 450.8 [(M + 2)-H]^−^, 452.9 [(M + 4)-H]^−^.

*2-(5-(3,4-Dichlorobenzylthio)-4-phenyl-4H-1,2,4-triazol-3-yl)-1H-indole* (**6f**). White powder m.p. 215 °C (yield 88%); IR (KBr): ν (cm^−1^) 3207 (NH), 3057 (C-H, aromatic), 2918 (C-H, aliphatic), 1597, 1566 (C=N), 1496, 1398, 700; ^1^H NMR (DMSO-*d*_6_) *ppm*: 4.41 (s, 2H, CH_2_), 5.59 (s, 1H, H-3), 6.95 (t, *J* = 7.7 Hz, 1H, H_ar_.), 7.13-7.15 (m, 1H, H_ar_.), 7.35-7.38 (m, 2H, H_ar_.), 7.43 (d, *J* = 8.4 Hz, 1H, H_ar_.), 7.47 (d, *J* = 7.7 Hz, 2H, H_ar_.), 7.58 (d, *J* = 8.4 Hz, 2H, H_ar_.), 7.64-7.66 (m, 2H, H_ar_.), 7.68-7.70 (m, 2H, H_ar_.), 12.00 (s, 1H, NH); ^13^C NMR (DMSO-*d*_6_) *ppm*: 35.41 (-S-CH_2_), 101.9, 112.3, 120.2, 121.2, 123.6, 124.2, 127.6, 128.4, 129.8, 130.5, 130.7, 131.0, 131.2, 131.3, 131.6, 134.0, 136.9, 139.3, 149.9, 151.4 (C_ar_., CH_ar_.); MS *m/z* (ESI): 448.8 [M-H]^−^, 450.8 [(M + 2)-H]^−^, 452.5 [(M + 4)-H]^−^.

*2-(5-((4-Methylbenzyl)thio)-4-phenyl-4H-1,2,4-triazol-3-yl)-1H-indole* (**6g**): White powder m.p. 274 °C (yield 95%); IR (KBr): ν (cm^−1^) 3336 (NH), 3111 (C-H, aromatic), 2933 (C-H, aliphatic), 1647,1630 (C=N), 1436, 1344, 701; ^1^H NMR (DMSO-*d*_6_) *ppm*: 2.28 (s, 3H, CH_3_), 4.37 (s, 2H, CH_2_), 5.57 (s, 1H, H-3), 6.95 (t, *J* = 7.7 Hz, 1H, H_ar_.), 7.11-7.13 (m, 3H, H_ar_.), 7.22 (d, *J* = 7.7 Hz, 2H, H_ar_.), 7.35 (d, *J* = 7.7 Hz, 1H, H_ar_.), 7.40 (d, *J* = 7 Hz, 2H, H_ar_.), 7.43 (d, *J* = 8.4 Hz, 1H, H_ar_.), 7.63-7.65 (m, 2H, H_ar_.), 7.67-7.69 (m, 1H, H_ar_.), 12.00 (s, 1H, NH); ^13^C NMR (DMSO-*d*_6_) *ppm*: 21.1 (CH_3_), 36.9 (-S-CH_2_), 101.7, 112.3, 120.2, 121.2, 123.6,124.3, 127.6, 128.4, 129.4, 129.5, 130.6, 131.1, 134.1, 134.3, 136.9, 137.3, 149.6, 151.8 (C_ar_., CH_ar_.); MS *m/z*: 394.9 [M-H]^−^.

*2-(5-(Benzylthio)-4-(4-chlorophenyl)-4H-1,2,4-triazol-3-yl)-1H-indole* (**6h**): beige powder m.p. 245 °C (yield 70%); IR (KBr): ν (cm^−1^) 3147 (NH), 3089 (C-H, aromatic), 2924 (C-H, aliphatic), 1595, 1571 (C=N), 1494, 1436, 800, 701; ^1^H NMR (DMSO-*d*_6_) *ppm*: 4.41 (s, 2H, CH_2_), 5.67 (s, 1H, H-3), 6.96 (t, *J* = 7 Hz, 1H, H_ar_.), 7.15 (t, *J* = 7 Hz, 1H, H_ar_.), 7.27-7.29 (m, 1H, H_ar_.), 7.31-7.32 (m, 4H, H_ar_.), 7.41-7.44 (m, 2H, H_ar_.), 7.46 (d, *J* = 8.4 Hz, 2H, H_ar_.), 7.70 (d, *J* = 8.4 Hz, 2H, H_ar_.), 11.99 (s, 1H, NH); ^13^C NMR (DMSO-*d*_6_) *ppm*: 37.3 (-S-CH_2_), 102.0, 112.3, 120.2, 121.3, 123.6, 124.1, 127.6, 128.1, 128.9, 129.5, 130.4, 130.7, 133.0, 135.7, 136.9, 137.4, 149.5, 151.7 (C_ar_., CH_ar_.); MS *m/z*: 414.9 [M-H]^−^, 416.9 [(M + 2)-H]^−^.

*2-(5-(4-Nitrobenzylthio)-4-(4-chlorophenyl)-4H-1,2,4-triazol-3-yl)-1H-indole* (**6i**). Yellow powder m.p. 217 °C (yield 82%); IR (KBr): ν (cm^−1^) 3248 (NH), 3103 (C-H, aromatic), 2924 (C-H, aliphatic), 1598, 1471 (C=N), 1492, 1350 (NO_2_), 800, 701; ^1^H NMR (DMSO-*d*_6_) *ppm*: 4.53 (s, 2H, CH_2_), 5.69 (s, 1H, H-3), 6.96 (t, *J* = 7.7 Hz, 1H, H_ar_.), 7.15 (t, *J* = 7.7 Hz, 1H, H_ar_.), 7.42 (d, *J* = 8.4 Hz, 2H, H_ar_.), 7.55(d, *J* = 9.1 Hz, 2H, H_ar_.), 7.66 (d, *J* = 8.4 Hz, 2H, H_ar_.), 7.72 (d, *J* = 8.4 Hz, 2H, H_ar_.), 8.18 (d, *J* = 8.4 Hz, 2H, H_ar_.), 11.97 (s, 1H, NH); ^13^C NMR (DMSO-*d*_6_) *ppm*: 36.0 (-S-CH_2_), 102.0, 112.3, 120.2, 121.3, 123.7, 124.0, 127.6, 130.4, 130.7, 130.8, 132.9, 135.8, 136.9, 145.9, 147.2, 149.7, 151.2, 207.1 (C_ar_., CH_ar_.); MS *m/z*: 462.9 [M+H]^+^.

*4-((4-(4-Chlorophenyl)-5-(1H-indol-2-yl)-4H-1,2,4-triazol-3-ylthio)methyl)benzonitrile* (**6j**): Pale Yellow powder m.p. 190 °C (yield 67%); IR (KBr): ν (cm^−1^) 3354 (NH), 3091 (C-H, aromatic), 2920 (C-H, aliphatic), 2225 (CN), 1598, 1590 (C=N), 1498, 1454, 800, 701; ^1^H NMR (DMSO-*d*_6_) *ppm*: 4.48 (s, 2H, CH_2_), 5.69 (s, 1H, H-3), 6.96 (t, *J* = 7.7 Hz, 1H, H_ar_.), 7.15 (t, *J* = 7.7 Hz, 1H, H_ar_.), 7.43 (d, *J* = 8.4 Hz, 2H, H_ar_.), 7.54 (d, *J* = 8.4 Hz, 2H, H_ar_.), 7.58 (d, *J* = 7.7 Hz, 2H, H_ar_.), 7.70 (d, *J* = 7.70 Hz, 1H, H_ar_.), 7.73 (d, *J* = 9.1 Hz, 2H, H_ar_.), 7.79 (d, *J* = 7.7 Hz, 2H, H_ar_.), 11.97 (s, 1H, NH); ^13^C NMR (DMSO-*d*_6_) *ppm*: 36.0 (-S-CH_2_), 102.0, 112.3, 120.3, 121.4, 123.8, 124.1, 127.7, 130.4, 130.5, 130.8, 132.9, 133.1, 133.2, 133.7, 135.9, 136.8, 143.9, 149.6, 151.5 (C_ar_., CH_ar_., CN); MS *m/z*: 439.9 [M-H]^−^, 442 [(M + 2)-H]^−^.

*2-(5-(4-Chlorobenzylthio)-4-(4-chlorophenyl)-4H-1,2,4-triazol-3-yl)-1H-indole* (**6k**): Yellow powder m.p. 235 °C (yield 37%); IR (KBr): ν (cm^−1^) 3178 (NH), 3032 (C-H, aromatic), 2918 (C-H, aliphatic), 1490, 1400 (C=N), 1350, 800, 701; ^1^H NMR (DMSO-*d*_6_) *ppm*: 4.40 (s, 2H, CH_2_), 5.69 (s, 1H, H-3), 6.95-6.97 (m, 1H, H_ar_.), 7.15 (t, *J* = 7.7 Hz, 1H, H_ar_.), 7.37-7.40 (m, 4H, H_ar_.), 7.42-7.44 (m, 2H, H_ar_.), 7.52 (d, *J* = 8.4 Hz, 2H, H_ar_.), 7.72 (d, *J* = 8.4 Hz, 2H, H_ar_.) 11.99 (s, 1H, NH); ^13^C NMR (DMSO-*d*_6_) *ppm*: 36.2 (-S-CH_2_), 102.0, 112.3, 120.2, 121.3, 123.7, 124.1, 127.6, 128.9, 130.4, 130.8, 131.4, 132.6, 132.9, 135.8, 136.7, 136.9, 149.5, 151.5 (C_ar_., CH_ar_.); MS *m/z* (ESI): 450.8 [M-H]^−^, 452.7 [(M + 2)-H]^−^, 454 [(M + 4)-H]^−^.

*2-(5-(2,4-Dichlorobenzylthio)-4-(4-chlorophenyl)-4H-1,2,4-triazol-3-yl)-1H-indole* (**6l**): White powder m.p. 239 °C (yield 79%); IR (KBr): ν (cm^−1^) 3155 (NH), 3091 (C-H, aromatic), 2922 (C-H, aliphatic), 1591, 1580 (C=N), 1490, 1388, 800, 701. 600; ^1^H NMR (DMSO-*d*_6_) *ppm*: 4.43 (s, 2H, CH_2_), 5.70 (s, 1H, H-3), 6.96 (t, *J* = 7.7 Hz, 1H, H_ar_.), 7.14-7.16 (m, 1H, H_ar_.), 7.40-7.42 (dd, *J* =8.4, 2.1 Hz, 1H, H_ar_.), 7.43 (d, *J* = 7.7 Hz, 2H, H_ar_.), 7.53-7.57 (m, 3H, H_ar_.), 7.64 (d, *J* = 2.1 Hz, 1H, H_ar_.), 7.72-7.73 (m, 2H, H_ar_.), 12.00 (s, 1H, NH); ^13^C NMR (DMSO-*d*_6_) *ppm*: 34.8 (-S-CH_2_), 102.13, 112.3, 120.2, 121.3, 123.7, 124.1, 127.6, 127.9, 129.5, 130.4, 130.4, 130.7, 132.9, 133.2, 133.7, 134.7, 135.8, 137.0, 149.8, 150.8 (C_ar_., CH_ar_.); MS *m/z* (ESI): 482.8 [M-H]^−^, 484.8 [(M + 2) + H]^+^, 486.8 [(M+4)-H]^−^, 488.7 [(M+6)-H]^−^.

*2-(5-(3,4-Dichlorobenzylthio)-4-(4-chlorophenyl)-4H-1,2,4-triazol-3-yl)-1H-indole* (**6m**): Beige powder m.p. 229 °C (yield 83%); IR (KBr): ν (cm^−1^) 3251 (NH), 3053 (C-H, aromatic), 2922 (C-H, aliphatic), 1620, 1597 (C=N), 1496, 1342, 800, 701, 600; ^1^H NMR (DMSO-*d*_6_) *ppm*: 4.39 (s, 2H, CH_2_), 5.70 (s, 1H, H-3), 6.96 (t, *J* = 7 Hz, 1H, H_ar_.), 7.15 (t, *J* = 7 Hz, 1H, H_ar_.), 7.36-7.38 (m, 1H, H_ar_.), 7.43 (d, *J* = 7 Hz, 2H, H_ar_.), 7.55-7.67 (m, 3H, H_ar_.), 7.67 (s, 1H, H_ar_.), 7.72-7.73 (m, 2H, H_ar_.), 12.00 (s, 1H, NH); ^13^C NMR (DMSO-*d*_6_) *ppm*: 35.7 (-S-CH_2_), 102.1, 112.3, 120.2, 121.3, 123.7, 124.1, 127.6, 129.8, 130.4, 130.5, 130.7, 131.0, 131.3, 131.5, 132.9, 135.8, 137.0, 139.2, 149.7, 151.3 (C_ar_., CH_ar_.,CCl_ar_); MS *m/z* (ESI): 482.8 [M-H]^−^, 484.8 [(M + 2) + H]^+^, 486.8 [(M+4)-H]^−^, 488.7 [(M+6)-H]^−^.

*2-(4-(4-Chlorophenyl)-5-((4-methylbenzyl)thio)-4H-1,2,4-triazol-3-yl)-1H-indole* (**6n**): Yellow powder m.p. 295 °C (yield 80%); IR (KBr): ν (cm^−1^) 3203 (NH), 3061 (C-H, aromatic), 2918 (C-H, aliphatic), 1685, 1577 (C=N), 1492, 1346, 800, 701; ^1^H NMR (DMSO-*d*_6_) *ppm*: 2.10 (s, 3H, CH_3_), 4.36 (s, 2H, CH_2_), 5.68 (s, 1H, H-3), 6.95-6.97 (m, 1H, H_ar_.), 7.12 (d, *J* = 7.7 Hz, 2H, H_ar_.), 7.15 (t, *J* = 7.7 Hz, 1H, H_ar_.), 7.21 (d, *J* = 8.4 Hz, 2H, H_ar_.), 7.42-7.46 (m, 4H, H_ar_.), 7.71 (d, *J* = 9.1 Hz, 2H, H_ar_.), 12.00 (s, 1H, NH); ^13^C NMR (DMSO-*d*_6_) *ppm*: 21.18 (CH_3_), 37.1 (-S-CH_2_), 101.9, 112.3, 120.2, 121.3, 123.7, 124.1, 127.6, 127.9, 129.5, 130.4, 130.7, 133.0, 134.2, 135.7, 136.9, 137.3, 149.5, 151.7, (C_ar_., CH_ar_.); MS *m/z*: 428.8 [M-H]^+^, 430.9 [(M + 2)-H]^−^

*2-(5-(Benzylthio)-4-cyclohexyl-4H-1,2,4-triazol-3-yl)-1H-indole* (**6o**): White powder m.p. > 300 °C (yield 84%); IR (KBr): ν (cm^−1^) 3448 (NH), 3138 (C-H, aromatic), 2939 (C-H, aliphatic), 1676, 1660 (C=N), 1571, 1460, 701; ^1^H NMR (DMSO-*d*_6_) *ppm*: 1.05-1.11 (m, 1H, cyclohexyl), 1.26-1.32 (m, 2H, cyclohexyl), 1.60 (d, *J* = 13.3 Hz, 1H, cyclohexyl), 1.76 (s, 4H, cyclohexyl), 1.97-2.03 (m, 2H, cyclohexyl), 4.34-4.38 (m, 1H, cyclohexyl), 4.56 (s, 2H, CH_2_), 6.75 (s, 1H, H-3), 7.07-7.09 (m, 1H, H_ar_.), 7.19-7.22 (m, 1H, H_ar_.), 7.28-7.30 (m, 1H, H_ar_.), 7.33-7.35 (m, 2H, H_ar_.), 7.43 (d, *J* = 7 Hz, 2H, H_ar_), 7.47 (d, *J* = 7.7 Hz, 1H, H_ar_.), 7.65 (d, *J* = 8.4 Hz, 1H, H_ar_.), 11.83 (s, 1H, NH); ^13^C NMR (DMSO-*d*_6_) *ppm*: 24.9, 25.7, 31.1(cyclohexyl), 37.7 (-S-CH_2_), 56.7 (cyclohexyl), 103.6, 112.3, 120.2, 121.3, 123.4, 124.2, 127.9, 128.0, 128.9, 129.5, 136.9, 137.4, 149.7, 149.8 (C_ar_., CH_ar_); MS *m/z*: 386.9 [M-H]^−^.

*2-(5-(4-Nitrobenzylthio)-4-cyclohexyl-4H-1,2,4-triazol-3-yl)-1H-indole* (**6p**): Yellow powder m.p. 255 °C (yield 59%); IR (KBr): ν (cm^−1^) 3165 (NH), 3090 (C-H, aromatic), 2931 (C-H, aliphatic), 1618, 1598 (C=N), 1517, 1346 (NO_2_), 1444, 1436, 701; ^1^H NMR (DMSO-*d*_6_) *ppm*: 1.08-1.14 (m, 1H, cyclohexyl), 1.28-1.34 (m, 2H, cyclohexyl), 1.62 (d, *J* = 13.3 Hz, 1H, cyclohexyl), 1.78-1.79 (m, 4H, cyclohexyl), 1.97-2.03 (m, 2H, cyclohexyl), 4.35-4.39 (m, 1H, cyclohexyl), 4.70 (s, 2H, CH_2_), 6.78 (s, 1H, H-3), 7.07-7.09 (m, 1H, H_ar_.), 7.20-7.22 (m, 1H, H_ar_.), 7.47 (d, *J* = 8.4 Hz, 1H, H_ar_.), 7.65 (d, *J* = 7.7 Hz, 1H, H_ar_.), 7.74 (d, *J* = 9.1 Hz, 2H, H_ar_), 8.21 (d, *J* = 8.4 Hz, 2H, H_ar_.), 11.83 (s, 1H, NH); ^13^C NMR (DMSO-*d*_6_) *ppm*: 24.9, 25.7, 31.1 (cyclohexyl), 36.5 (-S-CH_2_), 56.7 (cyclohexyl), 103.7, 112.3, 120.2, 121.3, 123.4, 124.0, 124.1, 127.9, 130.8, 136.9, 146.1, 147.2, 149.1, 150.1 (C_ar_., CH_ar_); MS *m/z*: 431.9 [M-H]^−^.

*4-((4-Cyclohexyl-5-(1H-indol-2-yl)-4H-1,2,4-triazol-3-ylthio)methyl)benzonitrile* (**6q**): White powder m.p. 235 °C (yield 56%); IR (KBr): ν (cm^−1^) 3192 (NH), 3057 (C-H, aromatic), 2970 (C-H, aliphatic), 2225 (CN), 1685, 1606 (C=N), 1436, 701; ^1^H NMR (DMSO-*d*_6_) *ppm*: 1.10-1.12 (m, 1H, cyclohexyl), 1.29-1.34 (m, 2H, cyclohexyl), 1.62 (d, *J* = 12.6 Hz, 1H, cyclohexyl), 1.78 (d, *J* = 10.5 Hz, 4H, cyclohexyl), 1.96-2.02 (m, 2H, cyclohexyl), 4.37-4.38 (m, 1H, cyclohexyl), 4.65 (s, 2H, CH_2_), 6.78 (s, 1H, H-3), 7.07-7.09 (m, 1H, H_ar_.), 7.20-7.22 (m, 1H, H_ar_.), 7.46 (d, *J* = 8.4 Hz, 1H, H_ar_.), 7.65-7.67 (m, 3H, H_ar_.), 7.82 (d, *J* = 8.4 Hz, 2H, H_ar_), 11.83 (s, 1H, NH); ^13^C NMR (DMSO-*d*_6_) *ppm*: 24.9, 25.7, 31.1 (cyclohexyl), 36.8 (-S-CH_2_), 56.7 (cyclohexyl), 103.6, 110.59 112.3, 119.2, 120.3, 121.4, 123.4, 124.1, 127.9, 130.5, 132.8, 136.9, 143.9, 149.1, 150.1 (C_ar_., CH_ar_); MS *m/z*: 411.9 [M-H]^−^.

*2-(5-(4-Chlorobenzylthio)-4-cyclohexyl-4H-1,2,4-triazol-3-yl)-1H-indole* (**6r**): White powder m.p. 250 °C (yield 85%); IR (KBr): ν (cm^−1^) 3176 (NH), 3055 (C-H, aromatic), 2931 (C-H, aliphatic), 1589, 1580 (C=N), 1494, 1438, 800, 701; ^1^H NMR (DMSO-*d*_6_) *ppm*: 1.07-1.13 (m, 1H, cyclohexyl), 1.28-1.34 (m, 2H, cyclohexyl), 1.61 (d, *J* = 13.3 Hz, 1H, cyclohexyl), 1.78 (d, *J* = 10.5 Hz, 4H, cyclohexyl), 1.97-2.03 (m, 2H, cyclohexyl), 4.35-4.39 (m, 1H, cyclohexyl), 4.56 (s, 2H, CH_2_), 6.79 (s, 1H, H-3), 7.07-7.09 (m, 1H, H_ar_.), 7.20-7.22 (m, 1H, H_ar_.), 7.41 (d, *J* = 8.4 Hz, 2H, H_ar_.), 7.47-7.48 (m, 3H, H_ar_.), 7.66 (d, *J* = 8.4 Hz, 1H, H_ar_), 11.83 (s, 1H, NH); ^13^C NMR (DMSO-*d*_6_) *ppm*: 24.9, 25.7, 31.1 (cyclohexyl), 36.7 (-S-CH_2_), 56.7 (cyclohexyl), 103.6, 112.3, 120.2, 121.3, 123.4, 124.2, 127.9, 128.9, 131.4, 132.6, 136.9, 137.0, 149.5, 149.9 (C_ar_., CH_ar_); MS *m/z* (ESI): 420.9 [M-H]^−^, 422.9 [(M + 2)-H]^−^.

*2-(5-(2,4-Dichlorobenzylthio)-4-cyclohexyl-4H-1,2,4-triazol-3-yl)-1H-indole* (**6s**): Yellow powder m.p. 200 °C (yield 60%); IR (KBr): ν (cm^−1^) 3288 (NH), 3078 (C-H, aromatic), 2927 (C-H, aliphatic), 1570, 1560 (C=N), 1467, 1436, 800, 750, 701; ^1^H NMR (DMSO-*d*_6_) *ppm*: 1.07-1.13 (m, 1H, cyclohexyl), 1.28-1.34 (m, 2H, cyclohexyl), 1.61 (d, *J* = 12.6 Hz, 1H, cyclohexyl), 1.77 (s, 4H, cyclohexyl), 1.95-2.01 (m, 2H, cyclohexyl), 4.34-4.39 (m, 1H, cyclohexyl), 4.61 (s, 2H, CH_2_), 6.79 (s, 1H, H-3), 7.07-7.09 (m, 1H, H_ar_.), 7.20-7.22 (m, 1H, H_ar_.), 7.42 (dd, *J* = 8.4, 2.1 Hz, 1H, H_ar_.), 7.47 (d, *J* = 7.7 Hz, 1H, H_ar_.), 7.55 (d, *J* = 8.4 Hz, 1H, H_ar_), 7.66-7.68 (m, 1H, H_ar_.), 7.70 (d, *J* = 2.1 Hz, 1H, H_ar_.), 11.84 (s, 1H, NH); ^13^C NMR (DMSO-*d*_6_) *ppm*: 24.9, 25.7, 31.2 (cyclohexyl), 35.5 (-S-CH_2_), 56.8 (cyclohexyl), 103.6, 112.3, 120.2, 121.3, 123.4, 124.1, 127.9, 129.5, 133.2, 133.8, 134.1, 134.8, 136.9, 148.9, 149.9 (C_ar_., CH_ar_); MS *m/z* (ESI): 454.9 [M-H]^−^, 456.8 [(M + 2)-H]^−^, 458.9 [(M + 4)-H]^−^.

*2-(5-(3,4-Dichlorobenzylthio)-4-cyclohexyl-4H-1,2,4-triazol-3-yl)-1H-indole* (**6t**): Yellow powder m.p. 202 °C (yield 53%); IR (KBr): ν (cm^−1^) 3186 (NH), 3086 (C-H, aromatic), 2935 (C-H, aliphatic), 1577, 1540 (C=N), 1494, 1436, 800, 760, 701; ^1^H NMR (DMSO-*d*_6_) *ppm*: 1.10-1.14 (m, 1H, cyclohexyl), 1.30-1.35 (m, 2H, cyclohexyl), 1.62 (d, *J* = 12.6 Hz, 1H, cyclohexyl), 1.79 (d, *J* = 11.2 Hz, 4H, cyclohexyl), 1.98-2.02 (m, 2H, cyclohexyl), 4.36-4.40 (t, *J* = 12.6 Hz, 1H, cyclohexyl), 4.57 (s, 2H, CH_2_), 6.79 (s, 1H, H-3), 7.07-7.09 (m, 1H, H_ar_.), 7.20-7.22 (m, 1H, H_ar_.), 7.45-7.47 (m, 2H, H_ar_.), 7.61 (d, *J* = 8.4 Hz, 1H, H_ar_.), 7.66 (d, *J* = 8.4 Hz, 1H, H_ar_), 7.75 (d, *J* = 2.1 Hz, 1H, H_ar_.), 11.84 (s, 1H, NH); ^13^C NMR (DMSO-*d*_6_) *ppm*: 24.9, 25.7, 31.1 (cyclohexyl), 36.09 (-S-CH_2_), 56.8 (cyclohexyl), 103.6, 112.3, 120.2, 121.4, 123.4, 124.1, 127.9, 129.9, 130.5, 131.0, 131.3, 131.6, 137.0, 139.3, 149.3, 150.0 (C_ar_., CH_ar_); MS *m/z* (ESI): 454.9 [M-H]^−^, 456.8 [(M + 2)-H]^−^, 458.9 [(M + 4)-H]^−^.

*2-(4-Cyclohexyl-5-((4-methylbenzyl)thio)-4H-1,2,4-triazol-3-yl)-1H-indole* (**6u**): Yellow powder m.p. 190 °C (yield 84%); IR (KBr): ν (cm^−1^) 3200 (NH), 3055 (C-H, aromatic), 2922 (CH, aliphatic), 1637,1640 (C=N), 1494, 1436, 800, 700; ^1^H NMR (DMSO-*d*_6_) *ppm*: 1.07-1.12 (m, 1H, cyclohexyl), 1.27-1.33 (m, 2H, cyclohexyl), 1.61 (d, *J* = 12.5 Hz, 1H, cyclohexyl), 1.77 (d, *J* = 11.2 Hz, 4H, cyclohexyl), 1.98-2.04 (m, 2H, cyclohexyl), 2.28 (s, 3H, CH_3_), 4.35-4.39 (m, 1H, cyclohexyl), 4.52 (s, 2H, CH_2_), 6.79 (s, 1H, H-3), 7.07-7.09 (m, 1H, H_ar_.), 7.15 (d, *J* = 7.7 Hz, 2H, H_ar_.), 7.19-7.22 (m, 1H, H_ar_.), 7.33 (d, *J* = 7.7 Hz, 2H, H_ar_.), 7.47 (d, *J* = 8.4 Hz, 1H, H_ar_), 7.66 (d, *J* = 8.4 Hz, 1H, H_ar_), 11.84 (s, 1H, NH); ^13^C NMR (DMSO-*d*_6_) *ppm*: 21.2 (CH_3_), 24.9, 25.7, 31.1 (cyclohexyl), 37.52 (-S-CH_2_), 56.7 (cyclohexyl), 103.5, 112.3, 120.2, 121.3, 123.4, 124.3, 128.0, 129.5, 129.6, 134.3, 136.9, 137.3, 149.7, 149.8 (C_ar_., CH_ar_); MS *m/z*: 400.9 [M-H]^−^.

### 3.3. Antimicrobial Evaluation

#### 3.3.1. Materials

Dimethyl sulfoxide (100%) was used to dissolve the standards ampicillin, fluconazole, and the tested compounds **6a-u** to give an initial concentration of 1 mg/mL.

#### 3.3.2. Organisms

All the tested strains bacterial and Candida in this study were provided from King Khaled University hospital, Microbiology Department. Bacterial strains were divided into Gram-positive bacteria namely *Staphylococcus aureus* (ATCC 25923), *Bacillus subtilis* (ATCC 6633). *Escherichia coli* (ATCC 25966). *Pseudomonas aeruginosa* (ATCC 27853) and *Salmonella* (LT2) as negative strains. *Candida tropicalis* (ATCC 66019), *Candida glabrata* (clinical isolates) and *Candida albicans* (ATCC10231) as fungal strains. All these microorganisms were pre-cultured on nutrient agar (Oxoid) and PDA (potato dextrose agar, Oxoid). Then, 0.5 McFarland turbidity of each microbial suspension was prepared in 5 mL nutrient broth tubes for the antibacterial assays (Agar well diffusion technique and MIC test).

#### 3.3.3. Agar Well Diffusion Technique

The antimicrobial activity of the tested compounds was determined using the agar well diffusion method. The prepared microbial suspensions were loaded on the surface of Mueller Hinton (Oxoid) plates using a sterile cotton swab. The agar surface was perforated with a sterile cork borer (6mm), and 100 µL of each compound (1000 µg/mL) were transferred into each well correspondingly. Plates were incubated aerobically for 18-24 h at 37 °C. Diameters of the inhibition zones were measured around each well and were recorded in mm as average of triplicate trials. DMSO was used as a negative control, while ampicillin and fluconazole were used as positive controls [38].

#### 3.3.4. MIC Test

The minimum inhibitory concentration of the synthesized compounds in the current study against the microbial strains was determined using the micro-broth dilution assay (MIC). The concentrations of the extracts used for MICs ranged from 100% (1mg/mL) to 0.25%. In brief, 100 µL of Mueller–Hinton broth (MH) was first loaded in polystyrene sterile flat-bottom 96-well plates; then, 100 µL of each compound in study was added, and two fold dilutions were performed until the 0.25% concentration is reached; finally, 50 µL of each of the bacterial suspensions was loaded respectively. Plates were incubated at 37 °C for 18-24 hrs, following incubation plates that were read at 600 nm with an Elisa reader (Biotech). The first column of the 96-well plate was loaded with only with Mueller Hinton broth; the bacterial suspension was a positive control and the last column was the negative control consisting of the medium broth (MH) and the extract correspondingly. The lowest concentration of compounds where no visible bacterial growth was observed and recorded after 24 h of incubation was considered as the MIC. The experiments were repeated in triplicate [39].

## 4. Conclusions

A series of indole-1,2,4-triazole molecular hybrids **6a-u** was synthesized and their structures were confirmed by different spectroscopic tools. Their in vitro antibacterial evaluation revealed that they possess good to moderate antibacterial activity against most of the tested Gram-negative strains with DIZ values in the range of 11-15 mm and MIC value around 250 µg/mL. Meanwhile, their antifungal evaluation showed higher activity especially against *Candida tropicalis* with MIC value as low as 2 µg/mL for most of the tested compounds. Moreover, compound **6f** bearing *N*-phenyl and 3, 4-dichlorbenzyl moieties revealed a potent growth inhibition zone of 26 mm in the DIZ assay and MIC value of 2 µg/mL against *Candida albicans*. In addition, the antibacterial and the antifungal activities results imply the significance of the *para* benzyl substituent.

## Figures and Tables

**Table 1:** Antibacterial activity of the target compounds **6a-u** against E. coli, Pseudomonas aeruginosa, Salmonella typhimurium, Staphylococcus aureus, and Bacillus subtilis.

	*E. coli*		*Pseudomonas aeruginosa*		*Salmonella typhimurium*		*Staphylococcus aureus*		*Bacillus subtilis*	
**Compound No.**	DIZ ± SD *	MIC (µg/mL)	DIZ ± SD *	MIC (µg/mL)	DIZ ± SD *	MIC (µg/mL)	DIZ ± SD *	MIC (µg/mL)	DIZ ± SD *	MIC (µg/mL)
**6a**	11 ± 1.06	1000	0	-	10 ± 0.00	1000	0	-	10 ± 0.70	1000
**6b**	11 ± 0.70	250	13 ± 7.50	250	10 ± 0.70	250	0	-	10 ± 2.12	250
**6c**	11 ± 2.12	250	14 ± 8.08	250	11 ± 2.88	250	0	-	9 ± 0.00	250
**6d**	10 ± 1.41	250	15 ± 8.66	250	11 ± 3.46	250	0	-	9 ± 0.00	250
**6e**	12 ± 1.41	250	0	-	11 ± 1.73	250	0	-	9 ± 0.00	500
**6f**	11 ± 0.70	500	0	-	10 ± 0.00	500	0	-	10 ± 0.70	250
**6g**	11 ± 0.70	250	10 ±5.50	250	10 ± 1.15	250	0	-	10 ± 0.70	250
**6h**	12 ± 1.41	250	10 ± 1.00	250	10 ± 0.57	500	9 ± 0.57	1000	0	-
**6i**	12 ± 2.12	250	11 ± 1.52	250	10 ± 0.70	250	9 ± 1.52	250	0	-
**6j**	14 ± 3.53	1000	12 ± 1.32	500	11 ± 0.35	500	11 ± 1.41	500	0	-
**6k**	14 ± 3.53	250	11 ± 0.57	250	10 ± 0.57	250	10 ± 1.41	250	0	-
**6l**	14 ± 3.53	250	10 ± 1.15	250	9 ± 0.00	250	11 ± 1.00	500	0	-
**6m**	12 ± 2.82	250	10 ± 0.57	250	10 ± 1.73	250	10 ± 0.57	250	0	-
**6n**	13 ± 2.12	500	12 ± 1.15	250	11 ± 1.00	250	11 ± 1.00	250	0	-
**6o**	11 ± 0.70	1000	13 ± 2.51	1000	11 ± 1.00	1000	0	-	0	-
**6p**	11 ± 0.00	500	12 ± 2.00	500	11 ± 2.00	500	0	-	0	-
**6q**	12 ± 1.06	500	12 ± 1.52	250	11 ± 1.15	250	0	-	0	-
**6r**	12 ± 0.70	250	13 ± 2.64	500	12 ± 1.52	250	0	-	0	-
**6s**	10 ± 1.41	250	14 ± 3.60	250	11 ± 1.52	500	0	-	0	-
**6t**	11 ± 0.00	500	13 ± 3.01	500	11 ± 1.73	500	0	-	0	-
**6u**	11 ± 1.41	1000	13 ± 2.00	1000	12 ± 1.15	1000	0	-	12 ± 1.00	250
**Ampicillin**	08 ± 0.57	500	24 ± 0.40	1000	14 ± 0.60	500	0	500	08 ± 0.28	500

* The arithmetic mean of the inhibition zone diameters in mean ± standard deviation. (-) not determined.

**Table 2:** Antifungal activity of the target compounds **6a-u** against *Candida albicans*, *Candida tropicalis,* and *Candida glabrata*.

	*Candida albicans*		*Candida tropicalis*		*Candida glabrata*	
**Compound No.**	DIZ ± SD *	MIC (µg/mL)	DIZ ± SD *	MIC (µg/mL)	DIZ ± SD *	MIC (µg/mL)
**6a**	26 ± 0.28	1000	13 ± 0.57	2	10 ± 0.57	1000
**6b**	26 ± 0.11	250	14 ± 1.52	2	10 ± 0.00	250
**6c**	26 ± 0.28	250	14 ± 2.08	2	10 ± 0.57	250
**6d**	27 ± 0.00	250	14 ± 3.46	4	10 ± 1.00	125
**6e**	26 ± 0.28	250	14 ± 3.21	4	11 ± 1.15	250
**6f**	26 ± 0.28	2	14 ± 2.00	4	10 ± 0.00	250
**6g**	26 ± 0.11	250	14 ± 1.15	4	10 ± 0.57	1000
**6h**	0	-	12 ± 0.57	2	0	
**6i**	0	-	11 ± 0.57	4	0	-
**6j**	0	-	11 ± 0.57	4	0	-
**6k**	0	-	10 ± 0.00	2	0	-
**6l**	0	-	10 ± 0.00	2	0	-
**6m**	0	-	10 ± 0.57	2	0	-
**6n**	0	-	11 ± 0.57	2	0	-
**6o**	11 ± 0.70	1000	11 ± 0.57	16	0	-
**6p**	10 ± 0.00	32	11 ± 0.57	4	0	-
**6q**	10 ± 0.70	32	11 ± 0.57	12.5	0	-
**6r**	10 ± 0.70	32	14 ± 0.57	2	0	-
**6s**	10 ± 0.00	250	12 ± 1.52	2	10 ± 0.57	62.5
**6t**	11 ± 0.70	250	11 ± 0.57	2	10 ± 0.57	32
**6u**	10 ± 0.00	1000	17 ± 0.28	2	13 ± 1.00	16
**Fluconazole**	07 ± 0.28	250	15 ± 0.11	1000	15 ± 0.57	1000

* The arithmetic mean of the inhibition zone diameters in mean ± standard deviation. (-) not determined.

## Data Availability

Data is contained within the article or Supplementary Materials.

## Supplementary Materials

The microanalysis data are available in Table S1 in the supplementary materials. ^1^HNMR and ^13^CNMR of compound **6d** are also available in the supplementary materials.

## Sample Availability

Samples of the synthesized compounds are available from the corresponding authors.
