# Supplementary material for: Design, Synthesis, and Antimicrobial Activity of Certain New Indole-1,2,4 Triazole Conjugates
Source: Molecules. 2021 Apr 15;26(8):2292. doi: 10.3390/molecules26082292 (PMC8071222; doi:10.3390/molecules26082292)
Supplement: Supplementary file 1 [file molecules-26-02292-s001.zip › molecules-1137294-supplementary.pdf]

Table S1. Elemental analysis of the target compounds **6a –u**.

Sample Code	M.W. g/mol	Molecular formula	Calculated CHN (%)	Found CHN (%)
**6a**	382.13	$C_{23}H_{18}N_4S$	C:72.22; H:4.74; N:14.65	C:71.97; H:4.66; N:14.53
**6b**	427.11	$C_{23}H_{17}N_5O_2S$	C:64.62; H:4.01; N:16.38	C:64.51; H:4.08; N:16.14
**6c**	407.12	$C_{23}H_{17}N_5S$	C:70.74; H:4.21; N:17.19	C:70.50; H:4.21; N:16.98
**6d**	416.09	$C_{23}H_{17}ClN_4S$	C:66.26; H:4.11; N:13.44	C:66.47; H:4.01; N:13.36
**6e**	450.05	$C_{23}H_{16}Cl_2N_4S$	C:61.20; H:3.57; N:12.41	C:60.93; H:3.62; N:12.17
**6f**	450.05	$C_{23}H_{16}Cl_2N_4S$	C:61.20; H:3.57; N:12.41	C:60.98; H:3.62; N:12.31
**6g**	396.14	$C_{24}H_{20}N_4S$	C:72.7; H:5.08; N:14.3	C:72.48; H:5.11; N:14.06
**6h**	416.09	$C_{23}H_{17}ClN_4S$	C:66.26; H:4.11; N:13.44	C:65.98; H:4.25; N:13.29
**6i**	461.07	$C_{23}H_{16}ClN_5O_2S$	C:59.8; H:3.49; N:15.16	C:60.07; H:3.54; N:14.86
**6j**	441.08	$C_{24}H_{16}ClN_5S$	C:65.23; H:3.65; N:15.85	C:65.14; H:3.87; N:15.62
**6k**	450.05	$C_{23}H_{16}Cl_2N_4S$	C:61.2; H:3.57; N:12.41	C:60.96; H:3.45; N:12.18
**6l**	484.01	$C_{23}H_{15}Cl_3N_4S$	C:56.86; H:3.11; N:11.53	C:56.71; H:3.2; N:11.36
**6m**	484.01	$C_{23}H_{15}Cl_3N_4S$	C:56.86; H:3.11; N:11.53	C:56.75; H:3.29; N:11.34
**6n**	430.1	$C_{24}H_{19}ClN_4S$	C:66.89; H:4.44; N:13.00	C:70.06; H:4.36; N:13.24
**6o**	388.17	$C_{23}H_{24}N_4S$	C:71.10; H:6.23; N:14.42	C:70.92; H:6.21; N:14.38
**6p**	433.16	$C_{23}H_{23}N_5O_2S$	C:63.72; H:5.35; N:16.15	C:63.88; H:5.43; N:16.00
**6q**	413.17	$C_{24}H_{23}N_5S$	C:69.71; H:5.61; N:16.94	C:69.53; H:5.85; N:16.87
**6r**	422.13	$C_{23}H_{23}ClN_4S$	C:65.31; H:5.48; N:13.25	C:65.25; H:5.61; N:13.16
**6s**	456.09	$C_{23}H_{22}Cl_2N_4S$	C:60.39; H:4.85; N:12.25	C:60.31; H:4.73; N:12.19
**6t**	456.09	$C_{23}H_{22}Cl_2N_4S$	C:60.39; H:4.85; N:12.25	C:60.35; H:4.69; N:11.98
**6u**	402.19	$C_{24}H_{26}N_4S$	C:71.61; H:6.51; N:13.92	C:71.48; H:6.67; N:14.05

PROTON DMSO {C:\Bruker\TOPSPIN} abari 10

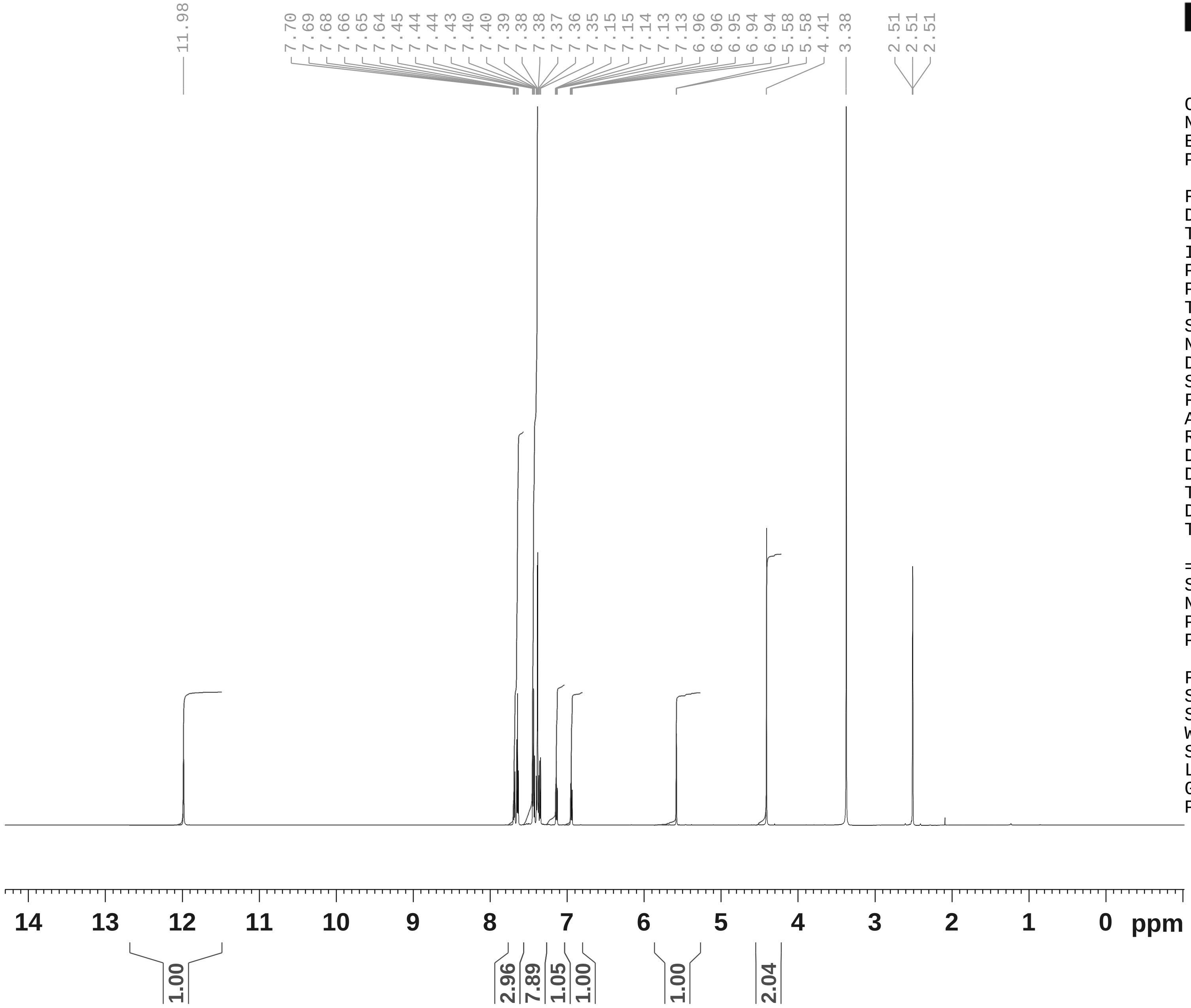

Current Data Parameters	
NAME	drMona-MM7
EXPNO	10
PROCNO	1
F2 - Acquisition Parameters	
Date_	20181011
Time	10.12
INSTRUM	spect
PROBHD	5 mm CPTCI 1H-
PULPROG	zg30
TD	65536
SOLVENT	DMSO
NS	16
DS	2
SWH	14097.744 Hz
FIDRES	0.215115 Hz
AQ	2.3243434 sec
RG	31.35
DW	35.467 usec
DE	31.86 usec
TE	295.0 K
D1	1.00000000 sec
TD0	1
======== CHANNEL f1 ========	
SFO1	700.1743238 MHz
NUC1	1H
P1	8.00 usec
PLW1	9.64999962 W
F2 - Processing parameters	
SI	65536
SF	700.1700000 MHz
WDW	EM
SSB	0
LB	0.30 Hz
GB	0
PC	1.00

C13CPD DMSO {C:\Bruker\TOPSPIN} abari 10

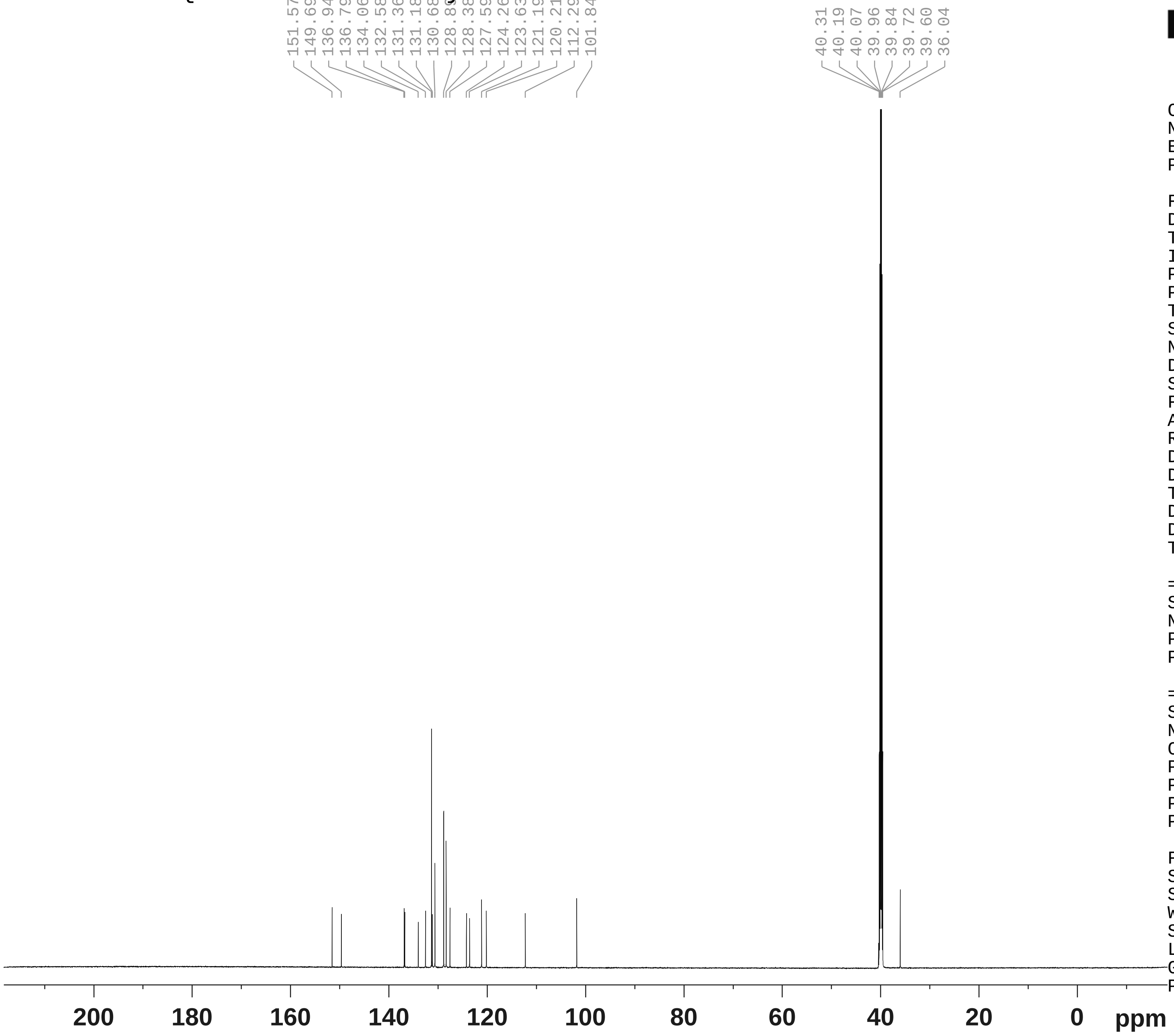


```
Current Data Parameters
NAME          drMona-MM7
EXPNO                 11
PROCNO                 1

F2 - Acquisition Parameters
Date_           20181011
Time               11.02
INSTRUM            spect
PROBHD   5 mm CPTCI 1H-
PULPROG          zgpg30
TD                 65536
SOLVENT             DMSO
NS                  1024
DS                     4
SWH           41666.668 Hz
FIDRES         0.635783 Hz
AQ            0.7864320 sec
RG                 172.3
DW                12.000 usec
DE                 18.00 usec
TE                 295.0 K
D1            2.00000000 sec
D11           0.03000000 sec
TD0                    1

======== CHANNEL f1 ========
SFO1        176.0754915 MHz
NUC1                 13C
P1                 12.00 usec
PLW1      121.00000000 W

======== CHANNEL f2 ========
SFO2        700.1728007 MHz
NUC2                  1H
CPDPRG[2         waltz16
PCPD2              65.00 usec
PLW2          9.64999962 W
PLW12         0.14618000 W
PLW13         0.06176000 W

F2 - Processing parameters
SI                 32768
SF          176.0578870 MHz
WDW                   EM
SSB      0
LB                  1.00 Hz
GB       0
PC                  1.40
```
